# Multiple Herpes Simplex Virus-1 (HSV-1) Reactivations Induce Protein Oxidative Damage in Mouse Brain: Novel Mechanisms for Alzheimer’s Disease Progression

**DOI:** 10.3390/microorganisms8070972

**Published:** 2020-06-29

**Authors:** Virginia Protto, Antonella Tramutola, Marco Fabiani, Maria Elena Marcocci, Giorgia Napoletani, Federica Iavarone, Federica Vincenzoni, Massimo Castagnola, Marzia Perluigi, Fabio Di Domenico, Giovanna De Chiara, Anna Teresa Palamara

**Affiliations:** 1 Department of Public Health and Infectious Diseases, Sapienza University of Rome, Laboratory affiliated to Istituto Pasteur Italia–Fondazione Cenci Bolognetti, 00185 Rome, Italy; virginia.protto@uniroma1.it (V.P.); marco.fab88@gmail.com (M.F.); mariaelena.marcocci@uniroma1.it (M.E.M.); giorgia.napoletani@uniroma1.it (G.N.); annateresa.palamara@uniroma1.it (A.T.P.); 2 Department of Biochemical Sciences, Sapienza University of Rome, 00185 Rome, Italy; antonella.tramutola@uniroma1.it (A.T.); marzia.perluigi@uniroma1.it (M.P.); fabio.didomenico@uniroma1.it (F.D.D.); 3 Istituto di Biochimica e Biochimica Clinica, Università Cattolica del Sacro Cuore, 00168 Rome, Italy; federica.Iavarone@unicatt.it (F.I.); federica.vincenzoni@unicatt.it (F.V.); 4 Fondazione Policlinico Universitario A. Gemelli, IRCCS, 00168 Rome, Italy; 5 Laboratorio di Proteomica e Metabolomica, IRCCS, Fondazione Santa Lucia, 00179 Rome, Italy; massimo.castagnola@icrm.cnr.it; 6 Istituto per la Chimica del Riconoscimento Molecolare, CNR, 00168 Rome, Italy; 7 Institute of Translational Pharmacology, National Research Council (CNR), 00133 Rome, Italy; 8 San Raffaele Pisana, IRCCS, Telematic University, 00163 Rome, Italy

**Keywords:** Herpes simplex virus-1, HSV-1, oxidative stress, redox proteomics, Alzheimer’s disease

## Abstract

Compelling evidence supports the role of oxidative stress in Alzheimer’s disease (AD) pathophysiology. Interestingly, Herpes simplex virus-1 (HSV-1), a neurotropic virus that establishes a lifelong latent infection in the trigeminal ganglion followed by periodic reactivations, has been reportedly linked both to AD and to oxidative stress conditions. Herein, we analyzed, through biochemical and redox proteomic approaches, the mouse model of recurrent HSV-1 infection we previously set up, to investigate whether multiple virus reactivations induced oxidative stress in the mouse brain and affected protein function and related intracellular pathways. Following multiple HSV-1 reactivations, we found in mouse brains increased levels of oxidative stress hallmarks, including 4-hydroxynonenal (HNE), and 13 HNE-modified proteins whose levels were found significantly altered in the cortex of HSV-1-infected mice compared to controls. We focused on two proteins previously linked to AD pathogenesis, i.e., glucose-regulated protein 78 (GRP78) and collapsin response-mediated protein 2 (CRMP2), which are involved in the unfolded protein response (UPR) and in microtubule stabilization, respectively. We found that recurrent HSV-1 infection disables GRP78 function and activates the UPR, whereas it prevents CRMP2 function in mouse brains. Overall, these data suggest that repeated HSV-1 reactivation into the brain may contribute to neurodegeneration also through oxidative damage.

## 1. Introduction

Alzheimer’s disease (AD), the most common form of dementia in the elderly [1] is a multifactorial disorder, likely resulting from the combination of different risk factors, including genetic, environmental, and, likely, infectious ones [2,3]. In addition to the pathological hallmarks of the disease, such as the accumulation of misfolded protein deposits in the brain as extracellular plaques containing amyloid β-peptides (Aβs) and intraneuronal neurofibrillary tangles formed by hyperphosphorylated tau proteins, AD brains exhibit evidence of reactive oxygen species (ROS)-mediated injury [4]. This includes modifications of the major components of cells, such as DNA, RNA, lipids, and proteins, occurring within oxidative stress conditions, i.e., when ROS production overcomes the antioxidant capability of the cells. Post-mortem analysis of AD brains showed increased levels of oxidative stress markers, such as protein 3-nitrotyrosine (3NT), protein carbonyls (PC), oxidized DNA bases, and lipid oxidation products [5]. In particular, lipid peroxidation is a specific product as well as a source of oxidative stress in the brain, since this tissue is characterized by the presence of high levels of polyunsaturated fatty acids (PUFA), the substrate for lipid peroxidation, a high rate of oxygen utilization, and low level of antioxidants [6]. This complex process implicates the reaction between oxygen-derived free radicals and PUFA, producing highly reactive electrophilic aldehydes that are capable of forming adducts with cysteine (Cys), lysine (Lys), or histidine (His) residues of proteins. Among these aldehydes, 4-hydroxynonenal (HNE) is one of the most abundant and toxic aldehydes generated through ROS-mediated peroxidation of lipids. Its toxicity relies on its capability to form Michael adducts on several proteins and, specifically, with the sulfhydryl group of Cys residues, the imidazole group of His residues, and the ε-amino group of Lys and arginine (Arg) residues. All these HNE-driven modifications may be detrimental to protein function and consequently to cells, thus HNE is considered a crucial player in oxidative injury of biomolecules. Interestingly, HNE–protein adducts together with increased levels of lipid peroxidation were detected in the brain of AD patients [6]. However, specific targets of lipid peroxidation-induced damage are yet to be fully explored.

A growing body of evidence suggests Herpes simplex virus-1 (HSV-1), a DNA virus able to establish a lifelong latent infection in sensory neurons with periodic reactivations, as one of the potential risk factors for AD. Starting from pivotal post-mortem studies showing the presence of HSV-1 DNA in those AD brains bearing the ε4 allele of apolipoprotein E, (a genetic risk factor for AD) [7,8], a recent multidisciplinary study [9], as well as population-based clinical ones [10,11,12,13,14,15] supported the hypothesis that periodic reactivations of the virus reaching the central nervous system (CNS) may predispose the brain to AD. In addition, several experimental studies, including ours, shed light on the possible mechanisms underlying HSV-1 detrimental effect in the CNS. In particular, we and others demonstrated the virus capability to induce in neuronal cells intra- and extracellular accumulation of Aβ peptide species [16,17,18,19] as well as their rapid fibrillization [20]. In addition, the virus was reported to trigger several neurotoxic pathways in neurons, including those related to the hyperphosphorylation/aggregation of the microtubule-associated tau protein [21] and synaptic dysfunction [22], the transcription of neurotoxic genes [23], and DNA damage [24]. Recently, by using a mouse model of recurrent HSV-1 infection, we showed the long-term dangerous effects of repeated HSV-1 replications in the brain (e.g., Aβ accumulation and deposition in plaques, tau hyperphosphorylation and aggregation, neuroinflammation), providing a first demonstration of the cause–effect relationship between HSV-1 reactivations and accumulation of AD molecular hallmarks, including cognitive deficits [25] and impaired adult neurogenesis [26]. Interestingly, HSV-1 life cycle is reported to induce an intracellular redox imbalance in host cells [27,28], and in vivo evidence demonstrated that this occurs also in the brain [29]. However, the effects of multiple virus reactivations in the brain in term of oxidative damage have yet to be clarified.

By taking advantage of the mouse model of recurrent HSV-1 infection [25], we addressed this issue in the present study, finding that multiple virus reactivations are paralleled by the accumulation of oxidatively modified proteins in mouse brains. A redox proteomic approach allowed us to identify HNE-modified proteins whose levels were significantly modulated in the cortex of HSV-1-infected mice as compared with those of mock-infected ones. Among them, we focused on glucose-regulated protein 78 (GRP78), a protein involved in the unfolded protein response (UPR) [30], and collapsin response-mediated protein 2 (CRMP2), a protein able to bind and stabilize microtubules [31]. We found that recurrent HSV-1 infection disables GRP78 function and activates UPR, whereas it prevents CRMP2 function in mouse brains.

## 2. Materials and Methods

### 2.1. Ethics Statement

The authors certify that all experimental protocols used in the present study are in agreement with the Italian and European legislation on animal experimentation (Decreto L.vo 26/2014; Direttiva n. 63/2010/EU). Experimental protocols were authorized by the Italian Ministry of Health (protocol numbers 801/2016-B, 745/2016-PR).

### 2.2. Mouse Model of Recurrent HSV-1 Infection

**Figure 1:**
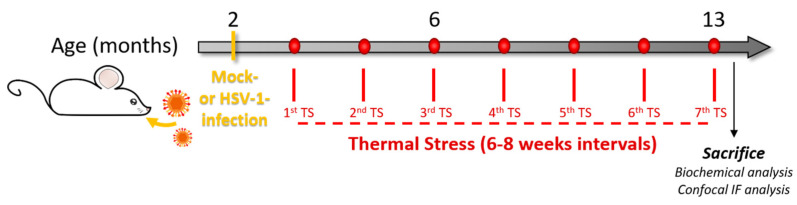
Experimental design. We infected two-month-old BALB/c mice with Herpes simplex virus-1 (HSV-1, HSV1-M) or a mock solution (CTRL-M). Six weeks after the infection, mice underwent several cycles of thermal stress (TS) that were spaced 6–8 weeks from each other. After the 7th TS, the mice were sacrificed, and brain tissues were collected for biochemical analyses (redox proteomics, Western blot, WB, and immunoprecipitation, IP) or perfused with paraformaldehyde (PFA) for confocal immunofluorescence analysis (IF).

We used brain tissue from the mouse model of recurrent HSV-1 infection we recently set up and characterized [25]. Briefly, 2 µl of HSV-1 suspension (equivalent to 10^6^ particle-forming units, pfu) or mock suspension were inoculated in 6–8-week-old female BALB/c mice (purchased from Harlan Laboratories) by lip scarification, following anesthesia with intraperitoneally injection of ketamine (80 mg/Kg) + xylazine (5 mg/Kg). HSV-1-infected mice (HSV1-M) and mock-infected ones (CTRL-M) were marked and kept in separate cages. When latent infection was established (6 weeks post-infection, p.i.) mice were individually subjected to hyperthermia (40–42 °C for 15 min: thermal stress = TS) to reactivate the latent virus, according to protocols from Sawtell et al. [32]. TS was repeated at 6–8-week intervals for 7 times during mouse life. Following the 7th TS, a set of animals per group was sacrificed and analyzed as described below (see experimental schedule in Figure 1). In this study, we used brain tissues from the mice assessed for AD hallmarks in De Chiara et al. 2019 [25]. For further details about the mouse model, see De Chiara et al. (2019) [25].

### 2.3. Western Blotting

Brain tissues (frontal cortex) from CTRL-M and HSV1-M (*n* = 6 per group) were homogenized by sonication on ice with RIPA buffer (20 mM Tris, 150 mM NaCl, 1% Triton X-100, 1% sodium deoxycholate, 0.1% SDS) supplied with protease and phosphatase inhibitor cocktails (Sigma-Aldrich, St Louis, MO, USA) and centrifuged at 15,000 g at 4 °C for 20 min to remove cellular debris. The supernatant was extracted to determine the total protein concentration by the the BCA method (Pierce, Rockford, IL, USA). Equal amounts of proteins (20 μg) were separated on SDS-PAGE using the TGX precast gels technology to obtain a Total Load gel used for the normalization of specific protein signals or using homemade 10% polyacrylamide gels. Subsequently, the gels were blotted onto nitrocellulose membranes (Amersham Protran 0.45 μm, Hybond ECL, GE Healthcare 0.22 μm, Chicago, IL, USA). The membranes were then blocked for 1–3 h in 10% non-fat milk or 3% bovine serum albumin (BSA, SERVA Electrophoresis GmbH, Heidelberg, Germany), as requested by the specific primary antibodies, in 0.1% tween-100 TBS (T-TBS) and incubated overnight at 4 °C with suitable dilutions of specific primary antibodies (in 5% non-fat milk or 3% BSA in T-TBS), such as anti-tubulin (1:5000, T6074, Sigma-Aldrich, St Louis, MO, USA), anti-HNE (1:2000, NB-10063093, Novus Biologicals, Centennial, CO, USA), anti-nitrotyrosine (1:1000, N0409, Sigma-Aldrich, St Louis, MO, USA), anti-GRP78 (1:500, SC-376768, Santa Cruz Biotechnology, Heidelberg, Germany), anti-CRMP2 (1:5000, 9393, Cell Signaling Technology, Danvers, MA, USA), anti-p-CRMP2 (1:1000, 9397, Cell Signaling Technology, Danvers, MA, USA), anti-p-PERK (1:1000, 3179S, Cell Signaling Technology, Danvers, MA, USA), anti-PERK (1:1000, SC-13073, Santa Cruz Biotechnology), anti-p-IRE1α (1:2000, NB100-2323, Novus Biologicals), anti- IRE1α (1:500, SC-390960, Santa Cruz Biotechnology, Heidelberg, Germany), and anti-ATF6 (1:1000, SC-166659, Santa Cruz Biotechnology, Heidelberg, Germany) antibodies. Secondary antibodies horseradish peroxidase-conjugated anti-mouse, anti-rabbit, or anti-goat IgG (1:10,000, Bio-Rad, Hercules, CA, USA) were incubated for 1 h at room temperature (RT). The membranes were developed with Clarity enhanced chemiluminescence (ECL) substrate (Bio-Rad, Hercules, CA, USA, #1705061), acquired using Chemi-Doc MP System (Bio-Rad, Hercules, CA, USA), and analyzed with Image Lab software (Bio-Rad, Hercules, CA, USA).

For carbonylated protein detection, after blocking, the membranes were equilibrated in 20% methanol, incubated in 2 N HCl, and finally derivatized with 0.5 mM 2,4-dinitrophenylhydrazone (DNP). Finally, DNP–protein adducts were detected on nitrocellulose using a primary DNP-specific antibody (MerckMillipore, Merck KGaA, Darmstadt, Germany). Following 3 washes in T-TBS, the membranes were incubated with horseradish peroxidase-conjugated antibodies (Jackson ImmunoResearch Laboratories, West Grove, PA, USA, 1:5000 in T-TBS). 

### 2.4. 2D Electrophoresis

Brain samples proteins (frontal cortex, 150 µg) from CTRL-M and HSV1-M (*n* = 6 per group) were isolated by trichloroacetic acid (TCA, 15% final volume) in ice for 10 min. Subsequently, the samples were centrifuged for 5 min at 10,000 g and the resulting pellets were rinsed three times in ice-cold ethanol/ethyl acetate 1:1 solution. The pellets were then resuspended in 200 µl of rehydration buffer (8 M urea, 20 mM dithiothreitol (DTT), 2.0% (*w*/*v*) Chaps, 0.2% Bio-Lyte, 2M thiourea, and bromophenol blue). Isoelectrofocusing (first dimension) was performed at RT using ReadyStrip™ IPG (11 cm, pH 3–10; Bio-Rad, Hercules, CA, USA) at 300 V for 2 h linearly, 500 V for 2 h linearly, 1000 V for 2 h linearly, 8000 V for 8 h linearly, and 8000 V for 10 h rapidly. Then, the isolectrofocusing strips were equilibrated in 50 mM Tris–HCl (pH 6.8) containing 6 M urea, 1% (*w*/*v*) sodium dodecyl sulfate (SDS), 30% (*v*/*v*) glycerol, and 0.5% DTT for 10 min and again for another 10 min in 50 mM Tris–HCl (pH 6.8) containing 6 M urea, 1% (*w*/*v*) sodium dodecyl sulfate (SDS), 30% (*v*/*v*) glycerol, and 4.5% iodoacetamide (IA). The electrophoresis step (second dimension) was performed using 12% acrylamide precast Criterion gels. The gels were then placed in fixing solution (7% acetic acid, 10% methanol) and incubated for 45 min. Afterward, the gels were stained for 2 h with SYPRO Ruby Gel Stain (Bio-Rad, Hercules, CA, USA) and finally de-stained overnight in deionized water. 

### 2.5. 2D Western Blot 

2D gels were then blotted onto nitrocellulose membranes (Bio-Rad, Hercules, CA, USA) and after 1 h incubation with 3% albumin in T-TBS to block unspecific binding sites, HNE–protein adducts were detected by 2 h staining at RT with a primary anti-HNE antibody (1:1000, NB-10063093 Novus Biologicals, Centennial, CO 80112, USA), followed by three washes with T-TBS and further incubation at RT for 1 h with a secondary alkaline phosphatase-conjugated anti-goat antibody (1:5000, Sigma-Aldrich, St. Louis, MO, USA). After extensively washes with T-TBS, the membranes were developed using a 5-bromo-4-chloro-3-indolyl phosphate/nitroblue tetrazolium solution (BCIP/NBT) prepared in alkaline phosphatase buffer solution.

### 2.6. Image Analysis

Redox proteomics gels and blots images were acquired by the Chemi-Doc MP System (Bio-Rad, Hercules, CA, USA) in TIFF format. Gels and blots (*n* = 12 each) were evaluated by PD-Quest 2D Analysis (7.2.0 version; Bio-Rad, Hercules, CA, USA). In brief, a master gel was chosen to identify spots on gels and blots, then spots from gels and blots were matched for normalization according to the total spot density. Gel-to-blot analysis was initially performed by manual matching of common spots and then, after obtaining a significant number of spots, by automated matching. Automated matching is created on user-defined parameters for spot detection based on the faintest spot, the largest spot, and the largest spot cluster that appear in the master gel. This process generates approximately 400–800 spots per gel or blot. Only proteins showing computer-determined significant (*p* < 0.05) differential levels between the groups (CTRL-M and HSV1-M) were considered for MS analysis. To define significant differences in proteins levels, analysis set manager software built in the PD-Quest software was used. The number of pixels that appear in a protein spot were calculated by the software, consistent with an increase or a decrease in protein levels. The image analysis was conducted in parallel on blots and on expression gels, and the results were then compared by the software to normalize HNE modification to expression value for each spot detected and matched. Data obtained by PD-QUEST software were analyzed using Student’s *t*-test. Significance was accepted if the *p* value was <0.05.

### 2.7. In-gel Trypsin Digestion/Peptide Extraction

Significantly different protein spots identified by the comparison between CTRL-M and HSV1-M were removed from the gels and individually transferred into microcentrifuge tubes for trypsin digestion. By using DTT and IA, disulphide bonds were reduced and cap and then gel plugs were incubated overnight at 37 °C with shaking in a trypsin solution. Tryptic peptides solutions were resuspended in water and stored at −80 °C for MS/MS analysis.

### 2.8. Reverse Phase -HPLC-High Resolution MS/MS Characterization of Tryptic Peptides

High-resolution HPLC–ESI–MS/MS experiments were performed using an Ultimate 3000 RSLC nano system coupled to an LTQ Orbitrap ELITE apparatus (Thermo Fisher Scientific, Waltham, MA, USA). Zorbax 300 SB-C18 (3.5 μm particle diameter; column dimension 1 mm × 150 mm) (Agilent Technologies, Santa Clara, CA, USA) was employed as the chromatographic column. The eluents used were: (A) 0.1% (*v*/*v*) aqueous FA and (B) 0.1% (*v*/*v*) FA in Acetonitrile (ACN)/water 80:20 *v*/*v*. The gradient applied was: 0–2 min 5% B, 2–40 min from 5 to 70% B (linear), 40–45 min from 70 to 99% B (linear), at a flow rate of 50 μL/min for a total of 65 min. MS spectra were collected with 120,000 resolution and m/z range from 350 to 2000. In data-dependent acquisition mode, the five most intense multiply charged ions were selected and fragmented in ion trap by using CID 35% normalized collision energy. Tuning parameters were: capillary temperature 300 °C, source voltage 4.0 kV.

### 2.9. MS Data Analysis

MS/MS data were analyzed by Proteome Discoverer software (version 1.4.1.14, Thermo Fisher Scientific, Waltham, MA, USA), based on SEQUEST HT cluster as search engine against UniProtKB mouse database (released on 28 of February 2017, *Mus musculus* 16,839 entries). Search parameters were: 10 ppm tolerance for precursor ions and 0.5 Da for product ions; 2 missed cleavage. Carbamydomethylation of cysteine (+57.02 Da) and oxidation of methionine (+15.99Da) were taken into consideration as fixed and variable modification, respectively. Protein characterization was set with the identification of a minimum of two peptides per protein and two unique peptides by applying the high-confidence filter. 

### 2.10. Immunoprecipitation

To validate the results obtained by MS, the identity of two specific proteins, GRP78– and CRMP2–HNE adducts, was evaluated using immunochemical methods. Samples (500 μg of proteins) were incubated overnight with specific antibodies (anti-GRP78 SC-13539, Santa Cruz Biotechnology, Inc., Heidelberg, Germany or anti-CRMP2 #9393, Cell Signaling Technology, Danvers, MA, USA) diluted in IP buffer (10 mM Tris, pH 7.6, 140 mM NaCl, 0.5% NP40 including protease inhibitors). The neoformed immunocomplexes were then incubated 2 h with Protein G beads (Sigma–Aldrich, St Louis, MO, USA) and then rinsed three times with RIA buffer (10 mM Tris, pH 7.6; 140 mM NaCl; 1% NP40). Proteins were separated by SDS-PAGE, and then the gels were immunoblotted onto nitrocellulose membranes (Bio-Rad, Hercules CA, USA). The membranes were incubated with the anti-HNE primary antibody followed by 1 h incubation with a peroxidase-conjugated secondary antibody (ImmunStar 1:10000, Bio-Rad, Hercules, CA, USA) or peroxidase-conjugated-Protein G (1:5000 Sigma–Aldrich, St Louis, MO, USA #18-161). Chemiluminescence was detected with Clarity enhanced chemiluminescence (ECL) substrate (Bio-Rad, Hercules, CA, USA, #1705061), and membranes acquired with Chemi-Doc MP image system (Bio-Rad, Hercules, CA, USA) and analyzed using Image Lab software (Bio-Rad, Hercules, CA, USA). The IP results were normalized on the total amount of the proteins of interest.

### 2.11. Immunofluorescence and Confocal Microscopy

Mouse perfusion was performed as described [25], and brains were post-fixed for 24 h in 4% paraformaldehyde (PFA), followed by cryoprotection for 24 h in 30% sucrose in PBS. Coronal brain sections (40-μm-thick) were obtained using a vibratome (VT1000 S, Leica Microsystems, Wetzlar, Germany). Free-floating sections were permeabilized 15 min at RT with 0.5% Triton X-100 in PBS, blocked 2h at RT with 10% horse serum (HS), 0.2% Triton X-100 in PBS, and then incubated overnight at 4 °C with specific primary antibodies (anti-GRP78 SC-1051 Santa Cruz Biotechnology, Heidelberg, Germany; anti-CRMP2 9393 Cell Signaling Technology, Danvers, MA, USA) diluted in antibody buffer (5% HS, 0.2% Triton X-100 in PBS). Sections were rinsed with PBS and then incubated 90 min at RT with secondary antibodies coupled to Alexa Fluor^®^ 488 or Alexa Fluor^®^ 546 dyes (Thermo Fisher Scientific, Waltham, MA, USA) diluted 1:1000 in antibody buffer. After extensively washing with PBS, the sections were incubated 20 min at RT with 4′,6′-diamidine-2′-phenylindole dihydrochloride (DAPI, Thermo Fisher Scientific, Waltham, MA, USA) and finally mounted on coverslips with Fluoromount G (Thermo Fisher Scientific, Waltham, MA, USA). The stained sections were imaged with a confocal laser-scanning microscope (Leica SP5, Leica Microsystems, Wetzlar, Germany) under sequential mode, to avoid crosstalk between channels. Images were acquired at 20X or 63X magnification. Image analysis was performed by the Imaris Suite 7.4 software (Bitplane A.G., Zurich, Switzerland). Quantification of immunofluorescence was carried out by drawing regions of interest (ROIs) in specific brain areas of the slices (cortex) and quantifying the mean fluorescence intensity in the ROIs. For the production of figures, processing was done by using the Adobe Photoshop CS6 software (Adobe Systems Incorporated, San Josè, CA, USA).

### 2.12. Statistics

Statistical comparisons were performed with GraphPad 6.0 (Prism) by using Student’s *t*-test. Data are presented as mean ± standard error of the mean (SEM). The level of significance was set at 0.05. All the experiments were performed by operators blind to the study conditions.

## 3. Results

### 3.1. Recurrent HSV-1 Infection Induces Oxidative Modifications of Proteins in Mouse Cortices

**Figure 2:**
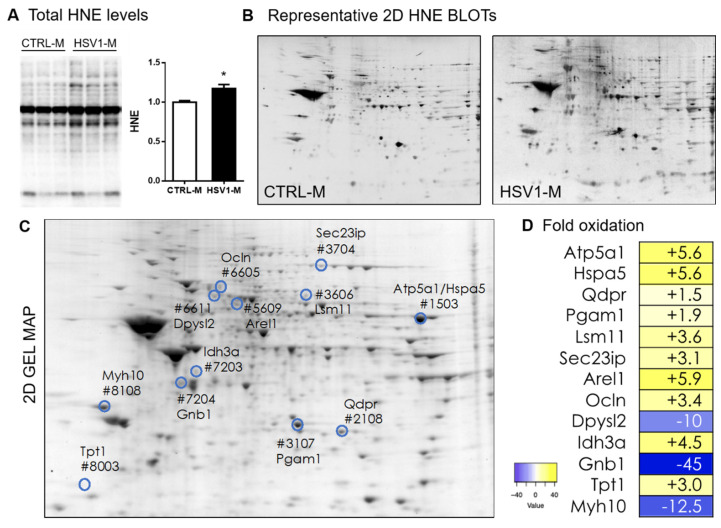
Total and protein-specific levels of 4-hydroxynonenal (HNE) in the brain of mice representing an in vivo model of recurrent HSV-1 reactivation. (**A**) Representative WB of HNE–protein levels in cortical lysates from HSV1-M or CTRL-M sacrificed after 7 cycles of TSs at 13 months of age. Densitometric analysis of oxidative modifications observed in HSV1-M (*n* = 6) are shown in the graph as fold increase compared to CTRL-M (*n* = 6). Each HNE densitometric value was normalized with total load within the same lane. Error bars represent SEM, (* *p* < 0.05 assessed by Student’s *t*-test). (**B**) Representative 2D-Map-blot proteomic profiles of HNE-oxidized proteins in cortical lysates from HSV1-M and CTRL-M sacrificed after 7 cycle of TS at 13 months of age. (**C**) Representative total protein spots detected in an HSV1-M sample by SYPRO Ruby staining in a 2D gel. Numbered spots indicate all the HNE-modified proteins with significant altered levels in HSV1-M with respect to CTRL-M. (**D**) Heat map and fold oxidation table of protein-specific HNE levels (see also Table 1).

We previously demonstrated that multiple HSV-1 reactivations reaching the brain induced hallmarks of neurodegeneration (i.e., accumulation of Aβ and phospho-tau and neuroinflammation) as well as cognitive deficits in mice [25], thus resembling the occurrence of an AD-like phenotype. Here, we investigated whether recurrent HSV-1 infections in mouse brain may induce also a progressive increment of oxidative stress conditions, as evidenced during the development of AD [33]. Specifically, we decided to investigate in this study the presence of oxidative damage in brain tissue of mice showing the most significant accumulation of AD hallmarks [25], which are a subset of mice that underwent seven cycles of TS-induced virus reactivation over their life and were sacrificed at 13 months of age (named hereafter HSV1-M, see experimental schedule in Figure 1). Hence, we first investigated the levels of oxidative stress markers in the brains of HSV1-M and matched mock-infected mice (named hereafter CTRL-M). In particular, we evaluated by western blot (WB) the level of oxidative modification of proteins indexed by the levels of HNE, 3-NT, and CP in cortical lysates from six HSV1-M and six CTRL-M. Results in Figure 2A and in Supplementary Figure S1 show that the levels of HNE (*p* = 0.01), 3-NT (*p* = 0.03), and CP (*p* = 0.03) were significantly increased in HSV1-M with respect to CTRL-M, indicating that recurrent HSV-1 infection triggers oxidative damage to proteins in mouse brains.

### 3.2. Recurrent HSV-1 Infection Induces an Altered HNE Profile in Mouse Cortices

We then employed redox proteomic analysis focusing on HNE modifications, since HNE levels and the levels of HNE–protein adducts are known to be increased in AD brains [6,34,35]. Specifically, we used a redox proteomic approach to identify protein targets of HNE peroxidation in cortices from the mice previously analyzed by WB for the levels of oxidatively modified proteins (see Figure 2). By comparing the densitometric intensities of individual spots of each HSV1-M sample with those measured in CTRL-M samples, we identified 12 HNE-modified spots whose levels were modulated (both upwards and downwards) in the cortex of HSV-1-M (see highlighted and numbered spots in Figure 2C). 

These spots were further analyzed by HPLC-ESI-MS/MS to identify the corresponding proteins. Specifically, in HSV1-M cortices, we found increased levels with respect to controls, ranging from 1.5- to 10-fold, of the HNE-protein adducts for dihydropteridine reductase (Dhpr), phosphoglycerate mutase 1 (Pgam1), translationally controlled tumor protein (Tpt1), occludin (Ocln), SEC23-interacting protein (Sec23ip), U7 snRNA-associated Sm-like protein LSm11 (Lsm11), osocitrate dehydrogenase [NAD] subunit alpha (Idh3a), ATP synthase subunit alpha (Atp5a1), glucose-regulated protein 78 (Hspa5, GRP78), and apoptosis-resistant E3 ubiquitin protein ligase 1 (Arel) (Table 1). Decreased levels, between 10- to 45-fold, were found for dihydropyrimidinase-related protein 2 (Dpysl2, CRMP2), myosin-10 (Myh10), and guanine nucleotide-binding protein G(I)/G(S)/G(T) subunit beta-1 (Gnb1).

### 3.3. STRING Analysis of Protein Networks

**Figure 3:**
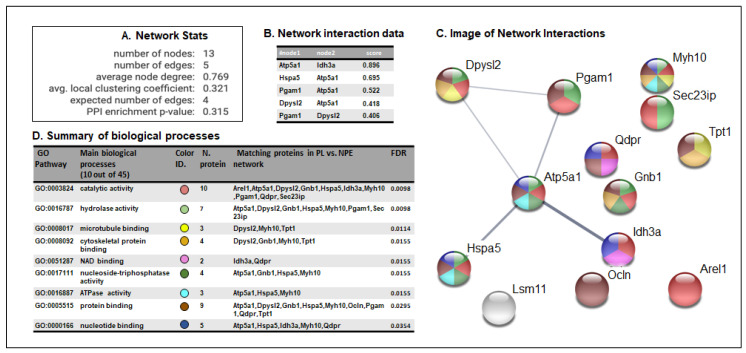
STRING analysis of proteomics data. (**A**) Network statistics reporting data concerning the number of nodes and edges, the average node degree, the average local clustering coefficient, the expected number of edges, and the protein-protein interaction (PPI) enrichment *p*-value. (**B**) Network interaction data table that reports all the significant interactions (min 0.4) between proteins of HSV1-M vs. those of CTRL-M; (**C**) Network interaction image showing nodes and edges between the proteins identified. The thickness of a line indicates the strength of the interaction between the proteins it connects. The colors of the spheres indicate the biological processes in which the proteins participate. (**D**) Biological process table reporting the main pathways in which each protein of the networks is involved. For each biological process identified, the corresponding Gene Ontology (GO) pathway, the number and identity of proteins, and the False Discovery Rate (FDR) are reported. The colors of the spheres indicate the biological processes in which the proteins participate.

The bioinformatics analysis of protein interaction networks and of biological processes for HNE-modified proteins in the comparison between HSV1-M and CTRL-M brains was built using the STRING software 11.0 [36,37]. This analysis allowed us to detect the biological repercussions of the aberrant HNE modification of the MS/MS-identified proteins. The analysis of the networks produced by the aberrantly oxidized proteins in the comparison between HSV1-M and CTRL-M (Figure 3A) showed a higher number of edges than expected (5 vs. 4), implying that the HNE-modified proteins interacted more strongly than what would be expected for a random set of proteins of similar size, drawn from the genome. Interaction networks are displayed in a table (Figure 3B) indicating interaction nodes and score and in an image showing shared biological processes (Figure 3C) which are fully elucidated in table D of Figure 3. Intriguingly, the majority of the proteins have catalytic, binding, or hydrolase activities, which are exploited in important cellular processes, such as energy metabolism (Dhpr, Pgam1, Idh3, and Atp5a1), protein folding (GRP78, Sec23ip), degradation processes (Arel1, Lsm11), and cell structure (Dpyl2, Gnb1, Myh10, Ocln, Tpt1), suggesting that their oxidative modification may deeply affect brain physiology, as previously demonstrated in AD [5,6].

### 3.4. GRP78 Focus

**Figure 4:**
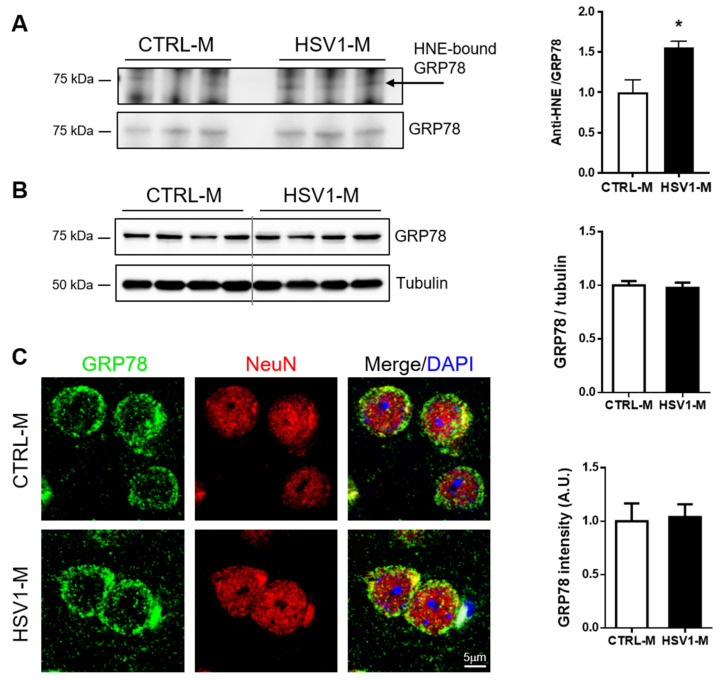
Multiple HSV-1 reactivations affect GRP78 oxidation but not its expression levels. (**A**): image and bar graph of the immunoprecipitation analysis of GRP78 HNE modification in HSV1-M compared to CTRL-M cortex samples (mean + SEM; * *p* < 0.05); (**B**): Representative western blot showing GRP78 expression in cortical homogenates from CTRL-M and HSV1-M. Tubulin expression level was used as sample loading control. Densitometric analyses of immunoreactive signals, normalized to tubulin levels, are shown in the graphs: values represent the normalized fold changes in protein levels from *n* = 6 HSV-M with respect to *n* = 6 CTRL-M (mean ± SEM). (**C**): Confocal immunofluorescence analysis of coronal brain slices from HSV1-M and CTRL-M (for each group, *n* = 3). Panels show representative neurons of the cortex that were immunostained for GRP78 (green) and NeuN (red). Cell nuclei were stained with DAPI (blue). Bar graphs show mean GRP78 fluorescence intensity quantified in the frontal cortex and expressed as fold change with respect to CTRL-M (mean ± SEM).

We next focused our attention on GRP78 (Hspa5), also known as BiP, that is a key protein in the UPR, a pathway involved in endoplasmic reticulum (ER) stress, and whose alterations are also linked to viral infection [30,38] and AD pathogenesis [39]. First, we validated the redox proteomic results by checking the levels of GRP78–HNE adducts in mouse cortical homogenates through an immunoprecipitation assay. In particular, by the aid of specific antibodies, we immunoprecipitated GRP78 and revealed by western blot the HNE-modified protein. Representative results in Figure 4A show that the amount of the immunoprecipitated HNE–GRP78 was significantly higher in HSV1-M cortices as compared with those of CTRL-M (*p* = 0.0175). In addition, WB analysis of tissue homogenates revealed that GRP78 expression was similar in cortices from HSV1-M compared to those of matched CTRL-M (Figure 4B, full length WB showed in Figure S2). Accordingly, IF analysis of brain slices did not reveal any difference in the expression levels of GRP78 between HSV1-M and CTRL-M (Figure 4C). 

**Figure 5:**
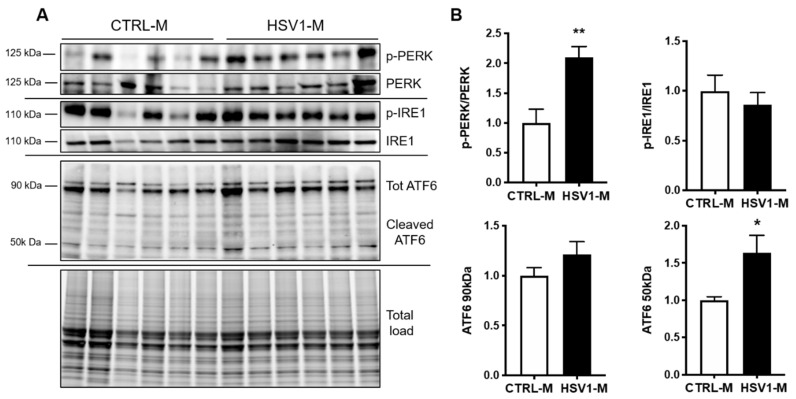
Multiple HSV-1 reactivation activates UPR. (**A**) Representative western blots showing total levels and phosphorylation of PERK and IRE1 and total and cleaved ATF6 levels in cortical homogenates from CTRL-M and HSV1-M. (**B**) Densitometric analysis of immunoreactive signals of phosphorylation/expression ratio normalized to total load is shown in the graphs: values represent the fold increase of HSV1-M with respect to CTRL-M, set to 1 (mean ± SEM); * *p* < 0.05; ** *p* < 0.02.

Next, since oxidative damage leads to GRP78 dysfunction and consequent aberrant activation of UPR, as previously observed in down syndrome (DS) [40,41], we investigated the activation of the three major arms of UPR pathways mediated by PERK, IRE, and ATF6. Specifically, we investigated changes in the levels of phospho-PERK (pT981, p-PERK) and phospho-IRE (pS724, p-IRE) and in the levels of ATF6 expression in cortical homogenates from HSV1-M and CTRL-M. Results in Figure 5A,B show that PERK activation (i.e., levels of pT981) was significantly increased in HSV1-M with respect to CTRL-M (*p* = 0.0038), whereas IRE phosphorylation was unaffected (*p* = 0.504). In addition, the levels of total ATF6 (90 Kd) were found unchanged following recurrent HSV-1 infection, whereas its active cleaved form (50 Kd) was found significantly increased in HSV1-M with respect to CTRL-M (*p* = 0.02). Full length WBs are shown in Figure S3. Overall, these data indicate that recurrent HSV-1 infection overactivates UPR in mouse brains, likely through GRP78 oxidation.

### 3.5. CRMP2 Focus

**Figure 6:**
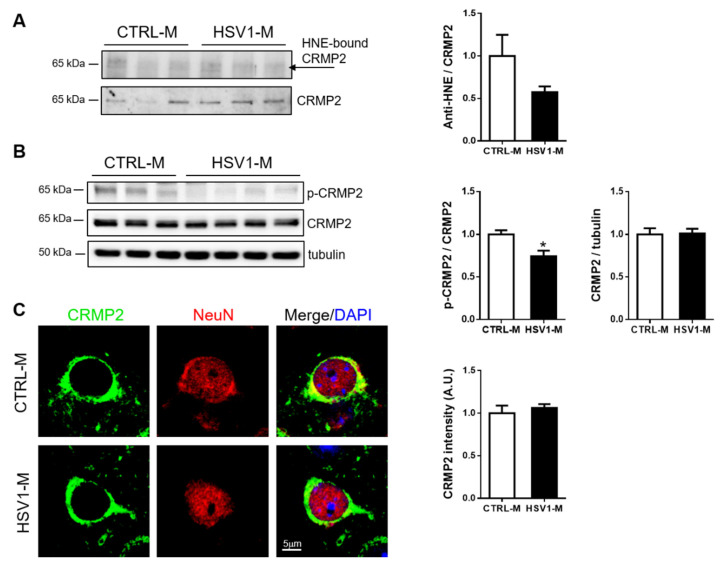
Multiple HSV-1 reactivations do not affect CRMP2 expression but inhibit its phosphorylation in T514. (**A**) Blots and bar graph of the immunoprecipitation analysis of CRMP2– HNE modification in HSV1-M compared to CTRL-M cortex samples; (**B**) Representative western blot showing the levels of CRMP2 phosphorylation at threonine 514 (p-CRMP2) and total CRMP2 expression in cortical homogenates from CTRL-M and HSV1-M. Tubulin expression level was used as a sample loading control. Densitometric analysis of immunoreactive signals normalized to CRMP2 (for p-CRMP2) or tubulin (for CRMP2) are shown in the graphs. Values represent the normalized fold changes in protein levels from *n* = 6 HSV-M with respect to *n* = 6 CTRL-M (mean ± SEM; * *p* < 0.05). (**C**): Confocal immunofluorescence analysis of coronal brain slices from HSV1-M and CTRL-M (*n* = 3). Panels show representative neurons of the cortex that were immunostained for CRMP2 (green) and NeuN (red). Cell nuclei were stained with DAPI (blue). Bar graphs show mean CRMP2 fluorescence intensity quantified in the frontal cortex and expressed as fold change with respect to CTRL-M (mean ± SEM).

Next, we focused our attention on Dpysl2, also known as CRMP2, since its oxidative modifications in mouse brains seemed to be prevented by multiple HSV-1 reactivation (10-fold decrease vs. CTRL-M, see Figure 4D and Table 1). CRMP2 is a ubiquitous protein in the brain and it is involved in microtubule stabilization, with a consequent regulatory role in cytoskeleton dynamics, neurite outgrowth, vesicle trafficking, and synaptic transmission in the developing brain [42]. Its function in the adult brain is not fully understood, but CRMP2 has been associated with several neuropathologic conditions including AD, where it has been found collapsed in neurofibrillary tangles upon hyperphosphorylation on different sites, including Thr-514 [43]. Thus, we firstly validated the redox proteomic data by an immunoprecipitation assay performed with cortical homogenates from *n* = 3 HSV1-M and *n* = 3 matched control mice. Results in Figure 6A show a decreasing trend in the amount of the immunoprecipitated HNE–CRMP2 in HSV1-M cortices as compared with CTRL-M ones. Then, we evaluated CRMP2 expression levels in mouse cortices by western blot and immunofluorescence analyses, finding that multiple HSV-1 reactivations did not affect them (Figure 6B,C). Finally, we investigated CRMP2 phosphorylation level in T514 (p-CRMP2): we found that it was significantly decreased in HSV1-M cortices as compared to CTRL-M ones (*p* = 0.0108, Figure 6B). Full length WBs are shown in Figure S2. Altogether these data suggest that multiple virus reactivations preserve CRMP2 function in mouse brain.

## 4. Discussion

A growing body of evidence supports the role of HSV-1 in AD pathogenesis and progression [44]. We previously demonstrated that recurrent HSV-1 infection in mice triggers the accumulation of the main AD pathognomic hallmarks, including cognitive dysfunction [25]. Since oxidative stress has been implicated in many neurodegenerative disorders, including AD [45,46,47,48], and oxidative stress conditions are associated with HSV-1 infection in host cells [27,29], in the present study, we investigated whether recurrent HSV-1 infection triggers oxidative stress in mouse brain, thus affecting protein function and contributing to neurodegeneration. We revealed that multiple HSV-1 reactivations cause oxidative modification in proteins and lipids (HNE, 3-NT, and CP) in the mouse cortex, resembling what occurs in the progression of AD [49,50]. In particular, studies from Butterfield’s group showed that several proteins in the brain of mild cognitive impairment (MCI) and AD subjects are oxidatively modified compared with controls, and that these irreversible modifications interfere with their function [51,52]. Indeed, these authors identified oxidatively modified proteins involved in specific cellular pathways, including energy metabolism, axonal integrity, chaperone machinery, and antioxidant systems, that correlate with the pathology of MCI and AD [52,53].

Along this line, our redox proteomic approach allowed us to identify 13 HNE adduct proteins profoundly modulated by recurrent HSV-1 infection (Table 1): the majority of these proteins are involved in cellular processes such as energy metabolism (Dhpr, Pgam1, Idh3, and Atp5a1), protein folding (GRP78, Sec23ip), degradation process (Arel1, Lsm11), and cell structure (Dpyl2, Gnb1, Myh10, Ocln, Tpt1). These pathways have been linked to AD onset and progression [54]. Hence, our data support the hypothesis that oxidative stress conditions induced by HSV-1 reactivation can concur to the virus-induced neurodegeneration.

Among the identified HNE–protein adducts that we found increased in HSV1-M cortices, we focused our attention on GRP78 (Hspa5: 5.6-fold oxidation, see Figure 2D and Table 1) that is a master protein in UPR response, a pathway involved in ER stress. In particular, GRP78 is a molecular chaperone member of the heat shock protein 70 (Hsp70) family of proteins, which transiently binds proteins into the ER to facilitate their folding, assembly, and transport [55]. Normally, GRP78 binds the intracellular domains of the three main UPR stress sensors ATF6, PERK, and IRE1, thus keeping them in an inactive state. Under ER stress, GRP78 acts as a chaperone to assist the folding of proteins accumulated in the ER and thus releases the UPR stress sensors. These, in turn, following phosphorylative events (e.g., for PERK and IRE1) as well as proteolytic ones (e.g., for ATF6), activate intracellular pathways to restore protein homeostasis or, when the stress persists, to induce apoptotic cell death. Specifically, activated PERK (p-PERK) phosphorylates the alpha subunit of eukaryotic translation initiation factor (eIF2α), which in turn inhibits protein translation to reduce ER protein burden; activated ATF6 (i.e., the cleaved form) translocates to the nucleus to upregulate the transcription of chaperone proteins, including GRP78; activated IRE1 acts as an endoribonuclease to upregulate genes responsible for protein folding and ER-associated degradation (ERAD) [56]. Intriguingly, among these three arms of the UPR, PERK is the most commonly linked to neurodegenerative disease, and increased p-PERK and p-eIF2α associated with pathology hallmarks have been found in AD brain tissues [39,57,58]. Indeed, ER stress and UPR are reportedly associated with AD pathogenesis, due to the accumulation of misfolded proteins in the brain, including Aβ and phospho-tau [39,57,59,60], and their chronic activation has been recently suggested as a key feature of this disease [61]. Interestingly, some papers showed that GRP78 is involved in Aβ clearance [62,63,64,65]. For instance, GRP78 was demonstrated to interact with APP, reducing Aβ40 and Aβ42 secretion. This may influence APP metabolism by impairing its access to β- and γ-secretases within the ER/Golgi or by facilitating its correct folding [55]. On the other hand, Aβ oligomers have been shown to induce cell death by eliciting ER stress, also though GRP78 overexpression [66], suggesting the existence of a complex scenario where ER stress and UPR may play a key role in neurodegeneration. Similarly, controversial data associated GRP78 expression/activation with tau phosphorylation. Some in vitro data reported that overexpressed GRP78 is able to bind GSK-3β, enhancing its kinase activity and thus causing an increase in tau phosphorylation [67,68]. On the contrary, other studies linked increased levels of tau phosphorylation and oligomer formation to aberrant UPR activation and GRP78 downregulation [48,69]. Di Domenico and colleagues demonstrated that oxidative damage, including that caused by lipid peroxidation, may affect GRP78 capability to bind misfolded proteins, providing a plausible link between deficit of molecular chaperones clearance activity, accumulation of misfolded proteins, risk of cognitive decline, and neurodegeneration [70]. Accordingly, our results, showing an accumulation of HNE-modified GRP78 in those HSV-1-M that displayed the main accumulation of neurodegenerative hallmarks [25], suggest that the oxidation-induced impairment of GRP78 activity may play a role in the virus-induced neurodegenerative effects. Along this line, we also provided evidence of UPR activation by measuring the levels of PERK phosphorylation as well as at those of ATF6 cleavage. This may actually be due both to virus-induced accumulation of misfolded proteins and to its replication cycles in the brain. Indeed, it is well known that the rapid synthesis of viral proteins can elicit the UPR in host cells as a first defense against the infection [71], and several viruses have evolved mechanisms to modulate UPR signaling for their own benefit. For instance, HSV1 is known to elicit PERK activation in the early stage of the infection, followed by both virus-mediated eIF2α dephosphorylation and degradation of cellular mRNA to allow the switch on of the translation machinery for viral protein synthesis [72]. In the same directions are other in vitro studies reporting a complex cross talk between HSV-1 infection and UPR activation. For instance, GRP78 expression was found both up- and downregulated during HSV-1 infection, mainly depending on HSV-1 strain and stage of the infection [73]. Other authors reported that the virus disarms the UPR in the early phase of the infection, activating only the ATF6 pathway and leaving the GRP78 levels unchanged and IRE1 inactive, to prevent further activation of ERAD that may be detrimental to virus replication [74]. This is in line with our data showing activation of ATF6 but not of IRE1. However, some authors reported IRE1 activation in HSV-1-infected cells, even though they documented that the RNAse and the kinase domains of this protein exert opposite roles in HSV-1 replication, inhibiting or favoring virus replication, respectively [75]. As a matter of fact, the net results of UPR activation upon virus entry may depend on the ability of the virus to exploit UPR pathways to support the correct folding of viral proteins and prevent their degradation through ERAD [74]. Thus, also our data may result from a complex balance between the effects of repeated HSV-1 replication in the brain (including the accumulation of AD-like misfolded proteins) and the host innate response to the infection.

Actually, we found both positively and negatively modulated HNE adducts, suggesting that the presence of the virus may also preserve the oxidative status of some proteins. This seems the case of CRMP2 (Dpysl2), whose oxidation was found preserved following multiple cycles of virus replication in the brain (−10-fold oxidation in HSV1-M vs. CTRL-M, see Figure 2D and Table 1). CRMP2 is known to bind tubulin heterodimers and to act as a regulator of the microtubule network. Interestingly, post-translational modifications including SUMOylation, O-GlcNAcylation, oxidation, and phosphorylation strictly regulate these functions [31]. Furthermore, according to computational analyses of CRMP structure [76], phosphorylation events may act sinergically with a redox switch at specific thiols to regulate the protein activity. This is in line with previous data reporting that upon oxidation on Cys504, a disulphide-linked CRMP2 homodimer forms a transient complex with thirodoxin1, allowing the activation of glycogen synthase kinase-3β (GSK-3β) and in turn the phosphorylation at Thr514 [77]. Indeed, among the kinases responsible of CRMP-2 phosphorylation, there are GSK-3β and cyclin-dependent kinase-5 (CDK-5), both kinases being involved also in tau phosphorylation. Specifically, GSK-3β phosphorylates CRMP2 on Thr-509 and Thr-514 [43], upon CDK-5-mediated phosphorylation on Ser-522 [43,78]. Interestingly CRMP2 hyperphosphorylation at these sites has been documented in AD brains, underlining its strict analogies to tau [42,79]. Our results show that CRMP2 phosphorylation levels at Thr-514 were decreased in HSV1-M compared to CTRL-M. However, compelling evidence, including ours, demonstrated that the virus promotes GSK3β activation and tau hyperphosphorylation in in vitro and in vivo models of infection [21,22,80]. In addition, we previously showed the presence of an aggregated form of phosphorylated tau in cortical homogenates from mice which underwent repeated cycles of virus reactivation [25]. Thus, it is possible to speculate that the virus preferentially affects tau protein functions. In addition, CRMP2, beside its link with tau, exerts also different functions, being involved in vesicle transport, endosomal–lysosomal trafficking, and autophagy [42]. In particular, CRMP2 functions as a cargo adaptor to the molecular motors kinesin-1 [81] (for anterograde transport) and dynein [82] (for retrograde transport). It is well known that HSV-1 exploits the host cytoskeleton (actin and microtubule networks) to travel along the cytoplasm, either from the membrane to the cell body (retrograde transport, exploited upon virus entry to reach the nucleus for productive infection or latency establishment) or from the cell body to the plasmatic membrane (anterograde transport, exploited for the release of virus progeny outside the infected cells) [83]. Thus, it is likely that the virus preserves CRMP2 function to assure its travelling along axons at the early stages of virus-driven AD onset. Once AD-like features are built, it is possible that also CRMP2 may undergo the well-known post-translational modifications that affect its function. Such a possibility is further supported by previous studies demonstrating the increased oxidation of CRMP2 in non-HSV1-infected AD brain [84,85,86]. Further experiments are required to clarify CRMP2 role in HSV-1 infection in the brain.

Overall, our redox proteomic data suggest that recurrent HSV-1 infections perturb the host cellular environment, in particular UPR, cytoskeleton network, and the clearance machinery for oxidized proteins and DNA. These events may contribute to the progressive accumulation of neurodegenerative hallmarks, supporting the role of HSV-1 in AD pathogenesis.

## Figures and Tables

**Table 1:** Proteins with increased HNE modification found in HSV1-M by redox proteomics analyses.

Spot N°	Protein	Uniprot N°	Fold	Score	Coverage	Peptide	Mw	pI
1503	ATP synthase subunit alpha, mitochondrial OS = Mus musculus GN = Atp5a1 PE = 1 SV = 1 - [ATPA_MOUSE]	Q03265	5.6↑	24	21.7	11	59.7	9.19
	78 kDa glucose-regulated protein OS = Mus musculus GN = Hspa5 PE = 1 SV = 3 - [GRP78_MOUSE]	P20029		2.1	2.4	1	72.4	5.16
2108	Dihydropteridine reductase OS = Mus musculus GN = Qdpr PE = 1 SV = 2 - [DHPR_MOUSE]	Q8BVI4	1.5↑	4.75	18.67	3	25.6	78.1
3107	Phosphoglycerate mutase 1 OS = Mus musculus GN = Pgam1 PE = 1 SV = 3 - [PGAM1_MOUSE]	Q9DBJ1	1.9↑	6.58	21.26	4	28.8	7.81
3606	U7 snRNA-associated Sm-like protein LSm11 OS = Mus musculus GN = Lsm11 PE = 1 SV = 1 - [LSM11_MOUSE]	Q8BUV6	3.6↑	2	6.93	1	39.9	10.73
3704	SEC23-interacting protein OS = Mus musculus GN = Sec23ip PE = 1 SV = 2 - [S23IP_MOUSE]	Q6NZC7	3.1↑	2.66	2.1	1	110.7	5.94
5609	Apoptosis-resistant E3 ubiquitin protein ligase 1 OS = Mus musculus GN = Arel1 PE = 2 SV = 2 - [AREL1_MOUSE]	Q8CHG5	5.9↑	2.41	1.58	1	94.1	7.25
6605	Occludin OS=Mus musculus GN = Ocln PE = 1 SV = 1 - [OCLN_MOUSE]	Q61146	3.4↑	2.26	1.73	1	59	6.48
6611	Dihydropyrimidinase-related protein 2 OS = Mus musculus GN = Dpysl2 PE = 1 SV = 2 - [DPYL2_MOUSE]	O08553	10.0↓	2.78	1.75	1	62.2	6.36
7203	Isocitrate dehydrogenase [NAD] subunit alpha, mitochondrial OS = Mus musculus GN = Idh3a PE = 1 SV = 1 - [IDH3A_MOUSE]	Q9D6R2	4.5↑	5.6	9.84	3	39.6	6.73
7204	Guanine nucleotide-binding protein G(I)/G(S)/G(T) subunit beta-1 OS=Mus musculus GN = Gnb1 PE = 1 SV = 3 - [GBB1_MOUSE]	P62874	45↓	4.38	7.35	3	37.4	6
8003	Translationally-controlled tumor protein OS=Mus musculus GN = Tpt1 PE = 1 SV = 1 - [TCTP_MOUSE]	P63028	3.0↑	2.19	7.56	1	19.4	4.86
8108	Myosin-10 OS = Mus musculus GN = Myh10 PE = 1 SV = 2 - [MYH10_MOUSE]	Q61879	12.5↓	2.08	0.4	1	228	5.54

The table shows: Spot identification number of PD-Quest analysis; full name and acronym of identified proteins; Uniprot identification number; protein-bound HNE fold changes in HSV1-M mice compared to CTRL-M set as 1; identification score; peptides coverage; Number of identified peptides for each protein; theoretical molecular weight (Mw), and isoelectric point (pI). Arrows indicate positive (↑) or negative (↓) fold-change.

## Supplementary Materials

The following are available online at https://www.mdpi.com/2076-2607/8/7/972/s1, Figure S1: Densitometric analysis of total and protein-specific levels of protein 3-nitrotyrosine (3 NT) and protein carbonyls (PC) in the brain of mice representing an in vivo model of recurrent HSV-1 reactivation; Figure S2: Full-length images of western blots shown in Figure 4 and Figure 6; Figure S3: Full-length images of western blots shown in Figure 5.
