# Supplementary material for: Multiple Herpes Simplex Virus-1 (HSV-1) Reactivations Induce Protein Oxidative Damage in Mouse Brain: Novel Mechanisms for Alzheimer’s Disease Progression
Source: Microorganisms. 2020 Jun 29;8(7):972. doi: 10.3390/microorganisms8070972 (PMC7409037; doi:10.3390/microorganisms8070972)
Supplement: Supplementary file 1 [file microorganisms-08-00972-s001.pdf]

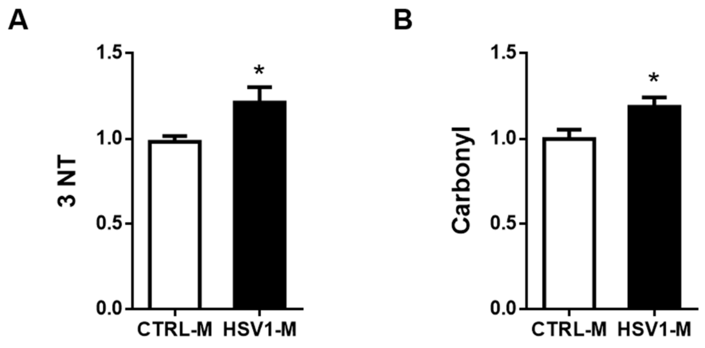

Figure 1. Densitometric analysis of total and protein-specific levels of protein 3-nitrotyrosine (3 NT) and protein carbonyls (PC) in the brain of mice representing an in vivo model of recurrent HSV-1 reactivation. Graphs showing the densitometric analysis of immunoblots for 3NT (**A**) and PC (**B**) protein levels in cortical lysates from HSV1-M or CTRL-M ($n = 6$ for each group) sacrificed after seven cycles of TS at 13 months of age. Data are shown as fold increase compared to CTRL-M. Error bars represent SEM, (* $p < 0.05$ assessed by Student's t-test).

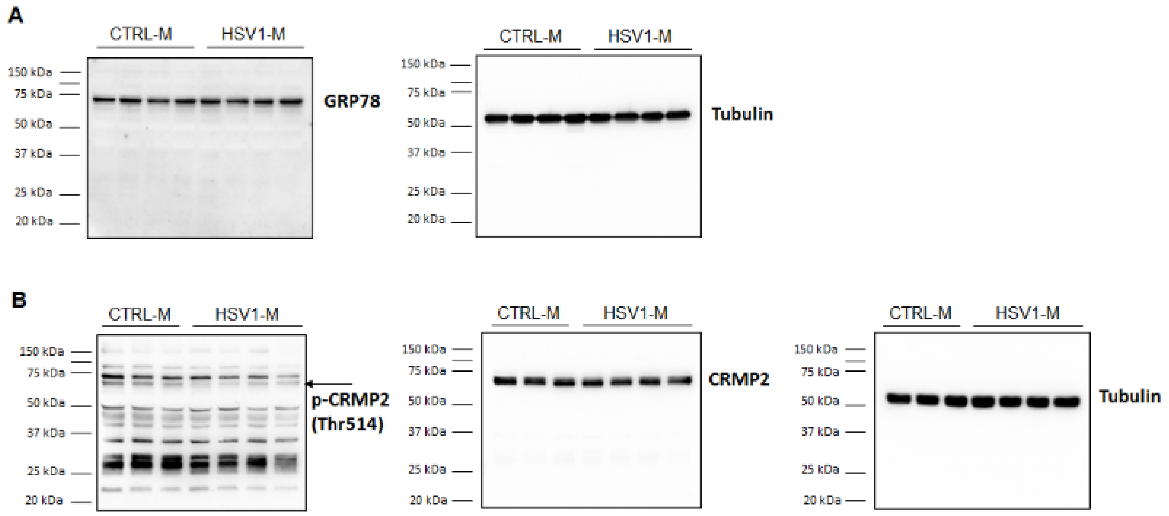

Figure 2. Full-length images of western blots shown in Figure 4 (**A**) and 6 (**B**). p-CRMP2 staining was performed after probing with other antibodies. The arrow indicates the specific band showing CRMP2 phosphorylation at Thr514.

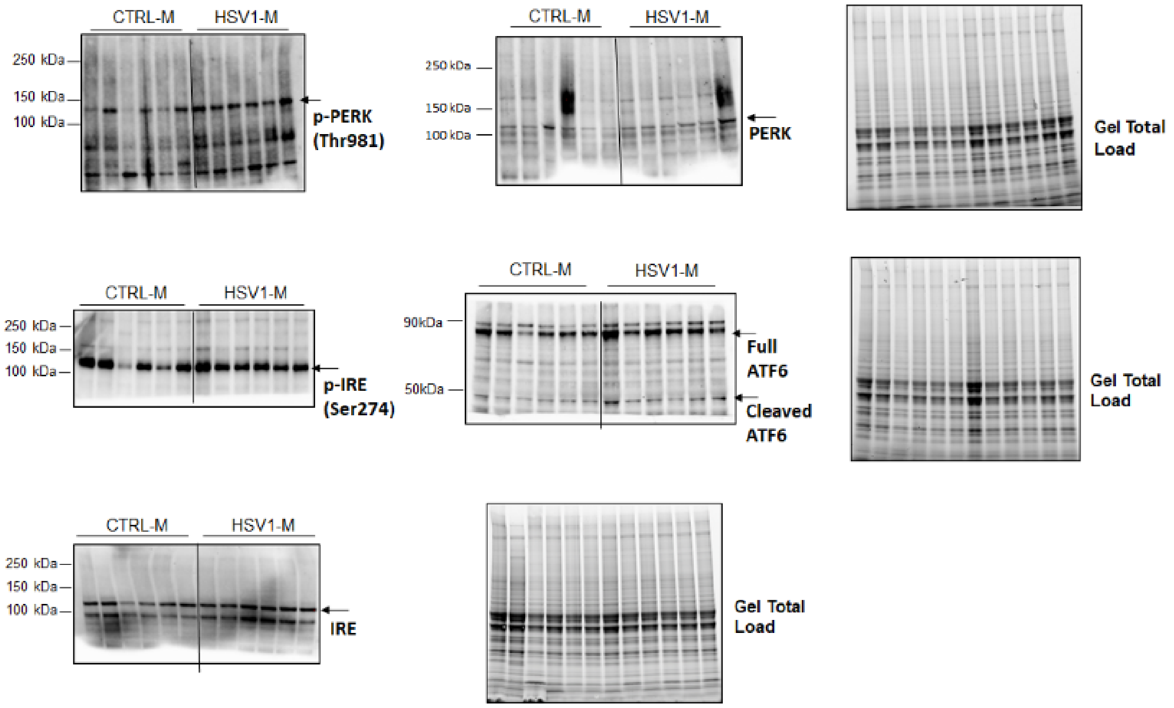

Figure 3. Full-length images of western blots shown in Figure 5.
